# A Multi-Species Simulation of Mosquito Disease Vector Development in Temperate Australian Tidal Wetlands Using Publicly Available Data

**DOI:** 10.3390/tropicalmed8040215

**Published:** 2023-04-03

**Authors:** Kerry Staples, Steven Richardson, Peter J. Neville, Jacques Oosthuizen

**Affiliations:** 1 Occupational and Environmental Health, School of Medical and Health Sciences, Edith Cowan University, Joondalup 6027, Australia; 2 School of Science, Edith Cowan University, Joondalup 6027, Australia; 3 Biological and Applied Environmental Health, Environmental Health Directorate, Department of Health, Perth 6849, Australia

**Keywords:** mathematical modelling, larval mosquito development, vector modelling, temperature-dependent development, *Aedes camptorhynchus*, *Aedes vigilax*, *Culex annulirostris*

## Abstract

Worldwide, mosquito monitoring and control programs consume large amounts of resources in the effort to minimise mosquito-borne disease incidence. On-site larval monitoring is highly effective but time consuming. A number of mechanistic models of mosquito development have been developed to reduce the reliance on larval monitoring, but none for Ross River virus, the most commonly occurring mosquito-borne disease in Australia. This research modifies existing mechanistic models for malaria vectors and applies it to a wetland field site in Southwest, Western Australia. Environmental monitoring data were applied to an enzyme kinetic model of larval mosquito development to simulate timing of adult emergence and relative population abundance of three mosquito vectors of the Ross River virus for the period of 2018–2020. The model results were compared with field measured adult mosquitoes trapped using carbon dioxide light traps. The model showed different patterns of emergence for the three mosquito species, capturing inter-seasonal and inter-year variation, and correlated well with field adult trapping data. The model provides a useful tool to investigate the effects of different weather and environmental variables on larval and adult mosquito development and can be used to investigate the possible effects of changes to short-term and long-term sea level and climate changes.

## 1. Introduction

Mosquitoes are present in environmental habitats ranging from the Arctic to the forests, deserts, and the tropics [1,2], and mosquito-borne disease is a global concern. The Ross River virus (RRV), present in Australia and the Western Pacific region, is the most commonly reported vector-borne disease in Australia, with 1451 to 9553 cases each year [3,4]. From the 1950s until 1987 when it was legally banned, Dichloro-Diphenyl-Trichloroethane (DDT) was used to control adult mosquitoes via broad spraying [5]. Current programs focus on controlling larval stages of mosquitoes, before they emerge as adults, using highly selective compounds such as s-methoprene and *Bacillus thuringiensis* subspecies *israelensis* (BTI). This involves larval monitoring, commonly conducted using a dipper at the larval habitat site [6]. To be effective and targeted, this type of monitoring requires routine observation of known larval habitats, which is time intensive and can only predict adult emergence a few days to a week in advance. Research into alternative ways of predicting adult mosquito populations and resultant disease, such as mathematical models, is needed. 

Since 2001 a range of statistical methods have been used to assess the potential environmental triggers that stimulate mosquito breeding cycles [7,8]. This has culminated in the identification of several important mosquito vectors including *Culex annulirostris* (Skuse), *Aedes vigilax* (Skuse), and *Aedes camptorhynchus* (Thomson) that are influenced by a range of environmental triggers including tides, river height, humidity, rainfall, sea-surface temperature, and air temperature. Subsequently models have been developed to predict epidemic outbreaks at local to regional levels but not at the larger state or national level [8,9,10,11,12,13,14,15,16,17,18,19].

To reduce the reliance of environmental health practitioners physically conducting larval monitoring and adult trapping to quantify mosquito activity, models that could be used to predict Ross River virus epidemics based on environmental variables were developed [14,19], thus negating the need for larval or adult mosquito surveillance. While this approach proved to be successful in many locations, the authors were not able to develop a statistically significant model for all areas studied. Most research to date highlights the need to incorporate mosquito abundance to increase the predictive power of these models [17,18,19,20,21,22,23]. 

Explicit models of environmental conditions and mosquito abundance have shown that adult *Ae. camptorhynchus* correlates with mean air temperature, *Ae. vigilax* correlates with mean air temperature, day length, tide height and tide frequency, and *Cx. annulirostris* correlates with site elevation and rainfall [13,24], but these environmental factors leave a large proportion of the variability in temporal abundance unexplained. Deterministic SEIR (Susceptible–Exposed–Infectious–Recovered) modelling has been used to investigate the relationship between RRV and the life traits of its mosquito vectors and found useful insights, but it did not encompass explicit larval components or the related real-time environmental inputs [20,25].

Statistical models together with adult trapping can provide a comprehensive analysis of disease threat under existing conditions but are unable to predict future events under changing habitats or environmental conditions. To investigate further, future scenario simulation models using known life traits of mosquito vectors would be useful for mosquito and mosquito-borne disease surveillance. Simulation models have been developed for malaria and dengue mosquito vectors [26,27,28,29] but not for Ross River virus vectors. 

To develop a useful simulation, a good understanding of the mosquito species under consideration is required. The mosquito lifecycle consists of four distinct phases: egg, larval, pupal, and adult, with the larval phase encompassing four instars. Active development of the sub-adult stages (egg, larval, and pupal) occurs in shallow water environments. The speed of development through these stages is regulated by temperature. The temperature response, or speed of development at a given temperature, varies across genera and species [30]. At any of the life stages, except the pupal stage, mosquitoes can enter a stage of dormancy (quiescence or diapause), although within-species dormancy mechanisms usually only exist at a single stage [31]. This interspecies variation makes it necessary to build species-specific representations to allow accurate estimation of timing and magnitude of adult mosquito emergence. 

Official description of Australian mosquitoes commenced in the late 19th century and research into their habitats and physiology gathered pace in the late 20th century [32], so while the total number of studies of RRV vectors is fewer than those focusing on malaria or dengue, a significant body of research has developed [7,33]. 

Ross River virus is transmitted by a wide range of mosquito species, the four most reported in Western Australia are *Ae. vigilax*, *Ae. camptorhynchus*, *Cx. annulirostris*, and *Ae. notoscriptus* [34]. These species can be characterized by their preferred larval habitats: saltwater and brackish tidal marshes (*Ae. camptorhynchus* and *Ae. vigilax*), permanent to semi-permanent freshwater sites (*Cx. annulirostris*), and small volume containers in close proximity to human populations (*Ae. notoscriptus)* [32,35]. This study investigates the dynamics of larger scale sites, which are suitable for the first three species.

*Aedes camptorhynchus* and *Ae. vigilax* are believed to be responsible for a high proportion of RRV transmission in Southwest, Western Australia [20,33,36]. Both emerge in high numbers from the tidal waters of the Swan River and are capable of dispersing many kilometers from the site of emergence [37,38].

Both species survive unfavourable environmental conditions via a diapausing egg stage. Their oviposition site preferences overlap with both preferring Samphire dominant vegetation habitats [39]. It has been theorized that *Ae. camptorhynchus* prefer sites in high tidal zones that are recharged by rainfall and groundwater and occasional very high tides in the cooler months, while *Ae. vigilax* prefer lower tidal areas, higher salinity levels, and emerge in higher numbers in the warmest months of the year [13]. The relationship between elevation and population density has resulted in mixed results for *Ae. vigilax* [24,40] and with vegetation being a stronger driver for *Ae. camptorhynchus* [41].

Eggs of *Ae. camptorhynchus* can remain viable for well over 12 months [42]. One report of the egg lifespan for *Aedes vigilax* is between 98 days at 17% relative humidity and 116 days at 65% [43]; however, the study methodology is unclear. Robust studies of other *Aedes* species have shown egg lifespans exceeding 200 days [44,45]. There is evidence that desiccation resistance is related to egg volume [31]. *Aedes camptorhynchus*, with a maximum egg survival of over 15 months have a larger egg volume than *Ae. vigilax* [46,47]; however, *Ae. notoscriptus* have an egg viability of over 1 year [44], despite a smaller egg volume [48]. It is possible the lifespan of *Ae. vigilax* eggs is significantly longer than previously reported.

Once laid, *Ae. vigilax* eggs take two days to complete embryonic development at 25 °C [49] while egg development time in *Ae. camptorhynchus* has not been studied. Other *Aedes* species have development times that vary with temperature. *Aedes aegypti* take 2 to 20 days to complete egg development [50], *Aedes albopictus* (Skuse) take 4.6 to 42 days at similar temperatures (Lee, 1994) as cited in [51], and *Aedes taeniorhynchus*, another wetland species, take 3 to 10 days at 20 to 27 °C [52]. It is likely *Ae. camptorhynchus* take longer than *Ae. vigilax* to complete egg development, in line with its other life traits, discussed below. *Aedes camptorhynchus* hatch over a wide range of temperatures and hatch in installments [53]. *Aedes vigilax* eggs hatch once a minimum temperature threshold is reached, and at warmer temperatures, all eggs hatch at once [43]. Once hatched, development and mortality are temperature dependent. *Aedes camptorhynchus* larvae develop into adults within 12 to 37 days [54]. *Aedes vigilax* can complete development in 5 to 20 days in the field and 5 to 14 days when held at constant temperatures in the laboratory [49,55].

*Culex annulirostris* are found in vegetated freshwater pools, ephemeral or permanent, including ponded streams, natural and constructed wetlands, and irrigation drains and have been shown to demonstrate vegetation preferences for oviposition Laird (1988) in [56] and elevation [24]. *Cx. annulirostris* have been found in the same larval habitats as *Ae. vigilax*, where it replaces *Ae. vigilax* as rainfall becomes the predominant environmental driver rather than brackish tides [32,57]. *Cx. annulirostris* can reproduce rapidly at temperatures above 25 °C [56] and disperse up to 10 km from its larval habitat [34]. Temperature-dependent development and mortality of all development stages of this species has been studied [56,58,59] in both laboratory and field conditions. Egg development in this species is very rapid and is completed within 1.25 to 5 days [58]. Adults can survive in laboratory conditions for up to 70 days [58]; however, field studies have estimated a 25 to 30 percent daily mortality rate, giving a mean survival time of 3–7 days [60]. At cooler temperatures *Cx. annulirostris* have been shown to enter a state of quiescence and overwinter as adults [61], and this period of dormancy and extended lifespan has been shown to be necessary to enable RRV transmission in this species [25].

There is sufficient understanding of the biology of these three mosquito species to enable a simulation model to be developed. A simulation model for mosquito development has been developed which includes separate hydrological and sub-adult compartments [27]. The sub-adult compartment consists of a set of equations describing the temperature-dependent development of malaria mosquito vectors. It includes a range of larval characteristics, including the effects of density-dependent mortality, sub-adult survival rates, and water temperature. Ross River virus mosquito vectors are different, and some parameters used for malaria may not be relevant. For example, *Cx. annulirostris* have been shown to be independent of density mortality [62]. Additionally, the literature for RRV vectors is not as comprehensive, so modification is required. The outputs from the sub-adult compartment of the malaria model include the number of adult mosquitoes. This sub-adult compartment was validated by [26] using a different hydrology compartment. The main driver of larval development is water temperature. Larval mosquito development responds non-linearly to temperature, so capturing the entire daily range of temperature fluctuation is important for accurately predicting mosquito development. Most temperature-dependent developmental studies are conducted under constant temperatures in the laboratory. It has been well established that mosquito larvae develop differently under constant versus varying temperatures, but the difference can be higher or lower depending on which part of the larval temperature range is encompassed [63,64,65,66,67,68].

The simulation model of [27] successfully predicts adult emergence but has some limitations including estimation of thermal mortality rates and underestimation of water temperature. The simulation model uses a thermal death point estimate to determine mortality due to high temperature where 10%, 50%, and 100% of larval mosquitoes die when temperatures of 1, 2, and 3 °C, respectively, above the thermal death point are reached. This does not account for accumulated mortality at less than lethal temperatures. Thermal mortality is thought to accumulate in larval mosquitoes. *Anopheles quadrimaculatus* larvae reared mostly at 25 °C have been found to occasionally emerge successfully at 35.5 °C, but larvae reared constantly at 35 °C would never emerge at that temperature [68]. 

The model described by [27] estimates water temperature using a model that assumes water temperature is always below air temperature. It is well documented that water temperature can be higher than air temperature for much of the day, due to the high thermal mass of water, taking longer to heat up and longer to cool down [69,70,71]. This small underestimate of temperature and therefore development, can accumulate over the immature mosquito stage. A shallow water temperature model that more accurately tracks water temperatures in a hot, humid region has been developed [72]. A modified version of which has been developed for use in Australian temperature conditions [73]. 

The aim of this research is to see if the current body of physiological development knowledge can be applied to simulate the local-scale pattern of emergence of vectors of Ross River virus in tidal wetland habitats using easily accessed environmental parameters such as tidal height, rainfall, temperature, and humidity. This simulation model should be capable of accurately predicting adult mosquito population patterns and assist in improving statistical models by inclusion of an entomological component without increasing the need for onsite mosquito surveillance and identification. The information provided would allow a more nuanced understanding of the dynamics of the mosquito populations within this habitat and may provide a means to estimate what may happen to mosquito species diversity and abundance under direct habitat modification, different mosquito control regimens, or as the local topography and climate change over the coming decades.

The study site, field sampling methods, and sources of public environmental data are outlined in the Materials and Methods section along with a diagram of the conceptual model. This is followed by a detailed explanation of the model equations used to represent the biological processes being simulated for temperature-dependent development and mortality at the egg, larval/pupal, and adult stages, and how these are applied specifically to each mosquito species. The number of adult female mosquitoes emerging each week is given in Section 3 and compared with on-site adult trapping data for validation. The model output for each mosquito species and the *Aedes* species egg bank is considered in the Discussion along with a reflection on the use and limitations of the model. 

## 2. Materials and Methods

This research applies an enzyme kinetic model of larval mosquito development from [27]. It incorporates hourly development and mortality estimates for sub-adult stages of *Cx. annulirostris*, *Ae. vigilax*, and *Ae. camptorhynchus* using the environmental variables river height, water depth, rainfall, humidity, windspeed, and air temperature. The outputs are compared with adult trapping data provided by the Local Government Authority and the Department of Health (WA) for model validation.

The mosquito development compartment is a set of equations describing the temperature-dependent development of mosquito vectors. It forms a loop of egg, larval/pupal, and adult development stages. The models are a set of iterative equations. The presence and temperature of water are the main drivers of mosquito development and mortality. Salinity, predation, nutrient limitation, and population density impacts are not included in the current model parameters. 

Larval and pupal stages are confined to the water, so each water body is considered as a point source of adult mosquito emergence. Hourly temperatures are used as larvae and pupae develop in shallow water which is homogeneous in temperature [73]. Adult mosquitoes can move to cooler or warmer sub-climates, such as under a shady tree, to avoid unfavourable conditions, so the daily mean air temperature is used for this stage. This is a closed-loop system in which all adults emerging and surviving to egg-laying stage will lay within these same waterbodies. There is no immigration of adults from other water sources for the *Aedes* species, but *Culex annulirostris* requires a stream of fecund adults as this species has no egg bank. 

The model runs for one year to cover the peak breeding season in the southern hemisphere (from January to December for *Ae. camptorhynchus*, and from July to June for the two remaining species) and loops generation by generation. *Culex* species were allowed to continue to loop until no more adults emerged. *Aedes* species were run for four or five generations until the pattern of emergence stabilized.

### 2.1. Study Site

**Figure 1:**
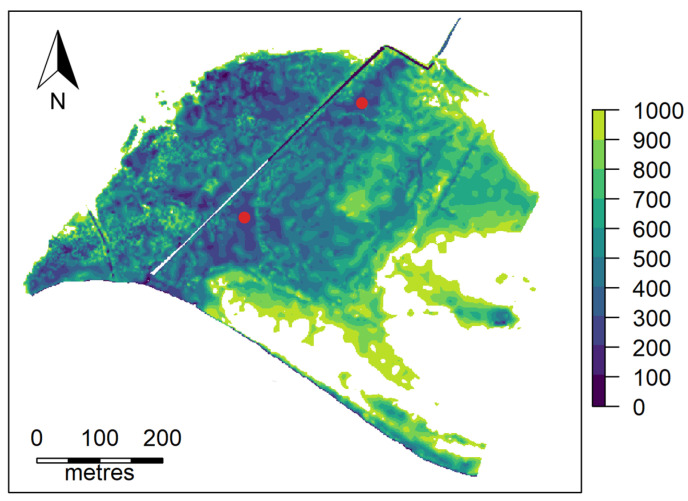
Ashfield Flats site map showing 100 mm contour break levels and the location of the two water bodies studied.

The study location is the Ashfield Flats, a tidal wetland adjacent to the Swan River in the suburb of Bassendean, located approximately 10 km east of Perth, Western Australia. The ground at the site is flat, varying from 0 to 400 mm in height (Australian Height Datum) over the main area of interest, Figure 1, and has an area of approximately 16 hectares. The surrounding land use is predominantly suburban residential. 

The area is subject to routine monitoring of larval, pupal, and adult stages using larval dippers and encephalitis virus surveillance carbon dioxide (CO_2_) light traps, respectively, and mosquito control chemicals are applied in response to larval mosquito activity. The primary chemical used in this area is the briquet formulation of s-methoprene, an insect growth regulator. As the area is bounded by residences, halting chemical intervention for the duration of this study would result in higher numbers of nuisance biting and increased risk of vector-borne disease in the surrounding area, and for this reason, the usual treatment and control practices were continued. 

Two tidal waterbodies within the Ashfield Flats site were modelled. Water height was measured at 15-min intervals, from August 2018 to November 2020, using staff gauges and capacitance probes. Initial water depth measurements, conducted by the Department of Biodiversity, Conservation and Attractions, were taken at 30-min intervals with a HOBO S-TMB-M006 temperature sensor from 11 September 2019 to 5 November 2019. Supplemental measurements were taken by the researcher using a LogTag UTRIX-16 temperature logger contained in a waterproof wrapping from 7 to 11 December 2021.

#### 2.1.1. Onsite Measurements

Sampling of the adult mosquito population using a carbon dioxide light trap (CO_2_ trap) [74] was conducted on 34 occasions by officers from the Local Government Authority as a part of their routine mosquito monitoring in the area. Where more than one trap was set on the same day, the highest trap count was used resulting in 32 trapping events over the three years analysed. 

#### 2.1.2. Public Data Sources

Long-term tidal heights for January 2018 to July 2021 were obtained for the Barrack Street Jetty [75]. Hourly air temperature, rainfall, humidity, wind speed, air pressure, and daily evapotranspiration and evaporation for the period from January 2018 to July 2021 were obtained from the Perth Airport weather station, located 2.5 km southeast of the study site [76].

### 2.2. Model Description

The model was coded utilizing R Studio Version 2022.02.3, using R version 4.2.0 and main packages; Matrix v 1.4-1, matrixStats v0.62.0, dplyr v1.0.9, zoo v1.8-10, and lubridate v1.8.0 (RStudio, PBC, Boston, MA, USA).

#### 2.2.1. Hydrology Compartment 

The hydrological compartment is a modification of [27] as detailed in [73]. Water levels from the Barrack Street Jetty were used as a proxy for river height at the study site by adding a vertical adjustment and meteorological variables from the Perth Airport weather station as inputs. The output of the hydrology compartment are water height and water temperature. These are used as inputs to the mosquito development compartment. The hydrology compartment has two main sections, water height and water temperature. 

The water height is calculated incrementally at one-hour intervals. A time vector t of length n is defined such that t(1) is the time at which the model is initiated, t(n) is the time that the model terminates, and $t\left(i+1\right)-t(i)$ equals one hour for all i=1,2…,n−1. Water height, $W_{H}(i)$, mm, denotes the water height at time, *t*(i) hours. Water height is determined as the balance of flows into and out of the site. Inflows include site-specific fixed inflows, $U_{IF}$, such as steams and pipelines, rainfall, $r_{a}$, and river height $R_{H}$ and $U_{IV}$ the water level increase per 1 mm of rainfall. Water outflows include water lost due to soil infiltration, $U_{O}$, and water lost due to evapotranspiration, ET which is scaled with a user-defined scale factor, $ET_{O}$. The river height impacts the water height only when a minimum overflow threshold, $R_{T}$, is exceeded, which represents the riverbank height adjacent to the wetland. All water flow variables have units of mm h^−1^, Equation (1).

(1) $$W_{H}\left(i+1\right)=\left\{\begin{array}{cc} \begin{array}{c} R_{H}\\ W_{H}\left(i\right)+U_{IF}+U_{IV}r_{a}-U_{O}-ET_{o}ET, \end{array}, & \begin{array}{c} R_{H}\geq R_{T}\\ R_{H}<R_{T} \end{array} \end{array}\right.$$

The water temperature is modelled using a one-layer iterative heat balance equation for shallow water pools developed by [77], with a modified calculation method for evaporative flux, LE, as detailed in [73], and shown in Equation (3). The change in water temperature is a function of incoming short wave, $K_{in}$, and long wave, $L_{in}$, radiation, and outgoing, $L_{out}$ radiation, allowing for solar reflection, $\alpha_{t}$, and heat exchange via convection at the water surface, H, heat exchange via evaporation at the water surface, LE, heat conduction at the soil/water interface, $G_{s}$, the density of water, $\rho_{w}$, the heat capacity of the water, $c_{w}$, the depth of water pool, $W_{H}$, and the water temperature at the previous time step, $T_{w}$, Equation (2).

(2) $$T_{w}\left(i+1\right)=T_{w}\left(i\right)+\frac{∆t\left(K_{in}\left(1-\alpha_{t}\right)+L_{in}-L_{out}-H-LE-G_{s}\right)}{\left(\rho_{w}c_{w}W_{H}\left(i\right)\right)}$$

**Figure 2:**
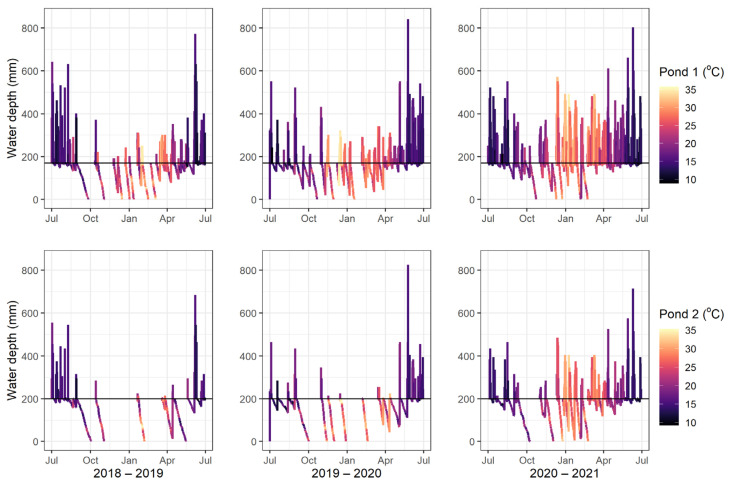
Water depth and daily mean water temperature for waterbody 1 (**top**) and waterbody 2 (**bottom**) for each year. The black line indicates the maximum depth of the pool once the river tide height recedes.

The hydrology compartment was run for 2018–2019, 2019–2020, and 2020–2021. The outputs for water temperature and water height are shown in Figure 2.

#### 2.2.2. Mosquito Development Model

**Figure 3:**
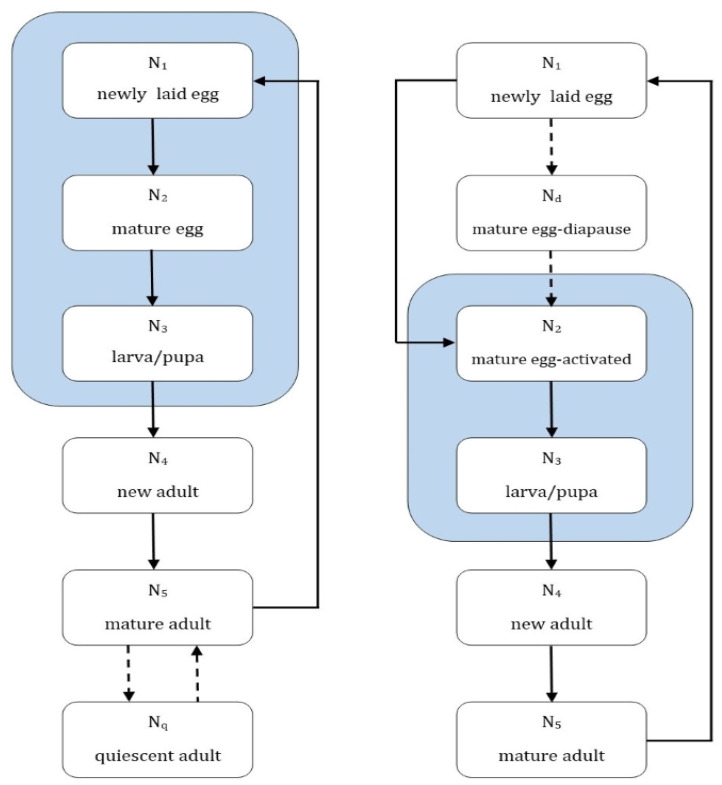
Mosquito development for *Culex annulirostris* (**right**) and *Aedes* spp. (**left**). $N_{i}$ denotes the stage number, and the blue shaded areas show stages dependent on the presence of water for completion. The dashed lines indicate an optional stage, which is triggered by unfavourable environmental conditions.

Parameter estimates are used to represent a “best-case” survival scenario for each mosquito species, Figure 3. This will over-estimate the mosquito population but will reveal the overall patterns of population growth and mortality. Due to the small number of studies of the physiology of the three Ross River virus mosquito vectors it was necessary to pool the larval and pupal stages so that they are treated as a single stage. Both larval and pupal stages occur in the aquatic environment and are affected similarly by water temperature, and this simplification reduces the computational complexity of the model while retaining fidelity to biological processes.

##### Temperature-Dependent Development

Egg and larval/pupal stages of development are determined by comparing the calculated cumulative development time, $CD_{t}$, of the mosquito to a mean value, $CD_{f}$, Equation (3). When the calculated cumulative development time exceeds the mean value, development is considered complete and the individual progresses to the next developmental stage. As some individual variation occurs in the field the $CD_{f}$ follows a normal distribution, and development is complete when:

(3) $$CD_{t}>CD_{f}+N\left(0,0.1CD_{f}\right)$$

The calculated cumulative development time is the sum of the development, $d_{k}$, at each time step, k, as shown in Equation (4).

(4) $$CD_{t}=\sum_{k=1}^{n}d_{k}$$

The development at each time step, $d_{k}$, for k=1,…,n, is determined over each time step, $∆t_{k}=t_{\left(k+1\right)}-t_{k}$, in hours, using the water temperature, $T_{w}$, and the development rate per hour, $r\left(T_{w}\right)$, as shown in Equation (5).

(5) $$d_{k}=r\left(T_{w}\right)∆t_{k}$$

The rate of development, $r\left(T_{w}\right)$, as shown in Equation (6), is governed by a temperature-dependent enzyme. The calculation requires estimates of the enthalpy of activation of the enzyme, $∆H_{A}^{\neq }$, and the change in enthalpy associated with low and high temperature inactivation of the enzyme $∆H_{L}$, and $∆H_{H}$, respectively. These values are estimated using curve fitting of data from observation of egg, larval, and pupal development. R is the universal gas constant and $\rho_{\left(25^{}{}^{\circ}C\right)}$ is the development rate per hour at 25 °C, assuming no inactivation of the enzyme, $T_{w}$ is water temperature, and $T_{\left(1/2H\right)}$ and $T_{\left(1/2L\right)}$ are the temperatures, in Kelvin, at which 50% of the enzyme is inactivated at the high and low temperatures, respectively, which were also determined using curve-fitting to experimental development rates as described in [78].

(6) $$r\left(T_{w}\right)=\frac{\rho_{25^{}{}^{\circ}C}\left(\frac{T_{w}+273}{298}\right)exp\left[\frac{∆H_{A}^{\neq }}{R}\left(\frac{1}{298}-\frac{1}{T_{w}+273}\right)\right]}{(1+exp\left[\frac{∆H_{L}}{R}\left(\frac{1}{T_{1/2L}}-\frac{1}{T_{w}+273}\right)\right]+exp\left[\frac{∆H_{H}}{R}\left(\frac{1}{T_{1/2H}}-\frac{1}{T_{w}+273}\right)\right]}$$

##### Temperature-Dependent Mortality

Larval/pupal mortality M is a cumulative sum of the hourly mortality at temperature, $T_{w}$, from the time the stage commences to the ith time step, Equation (7). Hourly mortality is estimated using curve-fitting of data from previous studies [52,54,58,79,80].

(7) $$M=\sum_{t_{l}}^{i}M\left(T_{w}\right)$$

Adult mosquito mortality, $M_{A}$ is a function of the time since emergence, t, and is estimated using observed data for each species where possible, as shown in Equation (8).

(8) $$M_{A}=A\ln\left(t\right)$$

Not all mosquito species and developmental stages have sufficient data available to develop consistent mortality and temperature-dependent development curves. Where required, species- and stage-specific modifications and alternate data sources are used for the egg and larval/pupal stages.

#### 2.2.3. Egg Stage

*Aedes* species’ newly laid eggs, $N_{1}$ and mature eggs $N_{2}$ can accumulate at the site and form an egg bank. To simulate this, the site is seeded with an equal number of mature eggs for each species at each contour height (mm). The initial number is estimated for both species using the density of *Ae. vigilax* eggs [81]. In contrast, *Culex* species lay eggs directly on the water surface and commence maturation and hatching without a period of dormancy. The model for this species commences with a population of adult females, laying five egg rafts per day, normally distributed with a standard deviation of 1. 

The three main characteristics of the egg stage are lifespan, development time, and hatching triggers: each being species specific, as shown in Table 1. 

##### Egg Survival

Survival for *Cx. annulirostris* eggs depend only on the presence of water, and they survive for up to 24 h if water is not present. Eggs are only laid when water is present. The egg survival proportion for *Ae. camptorhynchus* is dependent only on time, as shown in Equation (9).

(9) $$S\left(t\right)=\sum_{t_{l}}^{i}0.981\left(0.99982^{t}\right)$$

**Figure 4:**
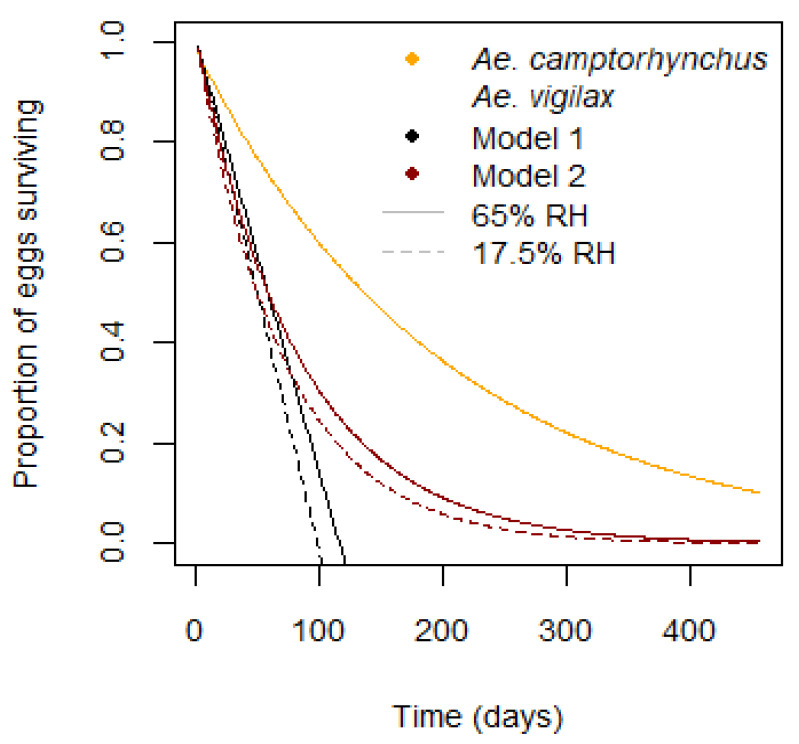
Egg survival by day for *Ae. camptorhynchus* and two models for *Ae. vigilax*. Model 1—linear, Model 2—proportional with equivalent median.

Egg survival in *Ae. vigilax* depends on relative humidity, *H.* Two models were tested, one linear, Equations (10) and (11), and one proportional, Equation (12). The first is an additive model, determined by adding the mortality at each timestep, $M_{i}$, from the time the egg is laid, $t_{l}$. Mortality is equal to the inverse of the lifespan at that humidity, $LE(H_{i})$. Mortality is summed to give a survival proportion giving a maximum egg viability of around 100 days. The second model takes the proportion surviving each day but multiplies it, with an adjustment so that the median value is equal to that of the linear model. This gives a longer maximum egg viability and better represents the real distribution of mosquito egg survival times [42,44]. These two survival models are shown for two humidity values, along with the value for *Ae. camptorhynchus,* in Figure 4.

(10) $$Mi=\frac{1}{{LE(H)}_{i}}$$

(11) $$S\left(t\right)=1-\sum_{t_{l}}^{i}M_{i}$$

(12) $$S\left(t\right)=\prod_{t_{l}}^{i}2exp\left(\frac{-2}{M_{i}}\right)$$

##### Egg Development and Hatching

**Figure 5:**
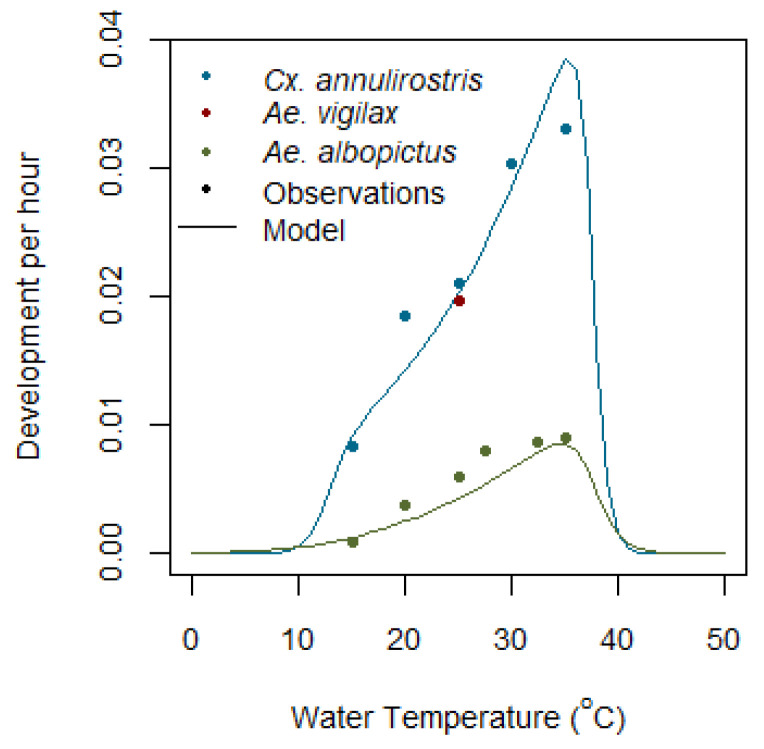
Modelled and observed egg development per hour for *Cx. annulirostris*, *Ae. vigilax*, and *Aedes albopictus* species.

In *Aedes* species, egg development depends on the air temperature, $T_{air}$, and is modelled using the rate of development equation given in Equation (6). After completing development, eggs remain dormant until they die (mortality, M, equal to one) or are submerged with water. If submerged, a proportion of eggs remaining viable hatch, and the rest are removed from the model. For *Cx. annulirostris,* egg development depends on the temperature of the water, $T_{w}$, as shown in Figure 5, [58] and is modelled using Equation (6).

*Aedes vigilax* egg development is completed in 48 to 54 h at 25 °C [49]. This figure is consistent with egg development in *Cx. annulirostris* at the same temperature and, in the absence of further data, the curve for *Cx. annulirostris* egg development is used for both species, as shown in Figure 5. Egg development time for *Ae. camptorhynchus* has not been studied, and egg development for *Aedes albopictus* is used as a proxy (Lee, 1994) in [51]. Once fully mature, eggs can hatch within the hour, subject to the correct hatching triggers (Standfast, 1967b in [57]).

*Aedes vigilax* eggs exhibit a minimum temperature threshold to commence hatching. Estimates of this vary: maximum air temperature is above 20 °C [82], or daily minimum temperatures above 11.5 °C [43]. Temperatures at the study site can easily exceed 20 °C all year round and have minimum temperatures above those of Sydney, where the previous studies occurred. Weekly mean temperature is less variable. Several hatching thresholds were tested. *Ae. camptorhynchus* does not have an explicit hatching threshold cited in the literature but a number were tested for this species.

The instalment hatching rate for *Ae. camptorhynchus* is 0.43 [53], and upon hatching there is a 17% hatch mortality [40]. *Aedes vigilax* exhibit some installment hatching, with 98% of mature eggs hatching when the water temperature exceeds 11.5 °C and none hatching if it is below 8 °C [43]. A linear instalment hatching proportion is applied for water temperatures between these two points.

The number of newly hatched larvae, $N_{3}$ is a function of the number of mature eggs, $N_{2}$, the survival proportion, S(t), the installment hatching proportion, $I_{H}$, and the hatch mortality, $M_{h}$, as shown in Equation (13).

(13) $$N_{3}=N_{2}S\left(t\right)I_{H}\left(1-M_{h}\right)$$

No hatch mortality rate has been estimated for *Ae. vigilax,* so the $H_{M}$ term is omitted from the equation.

#### 2.2.4. Larval/Pupal Development and Mortality

**Figure 6:**
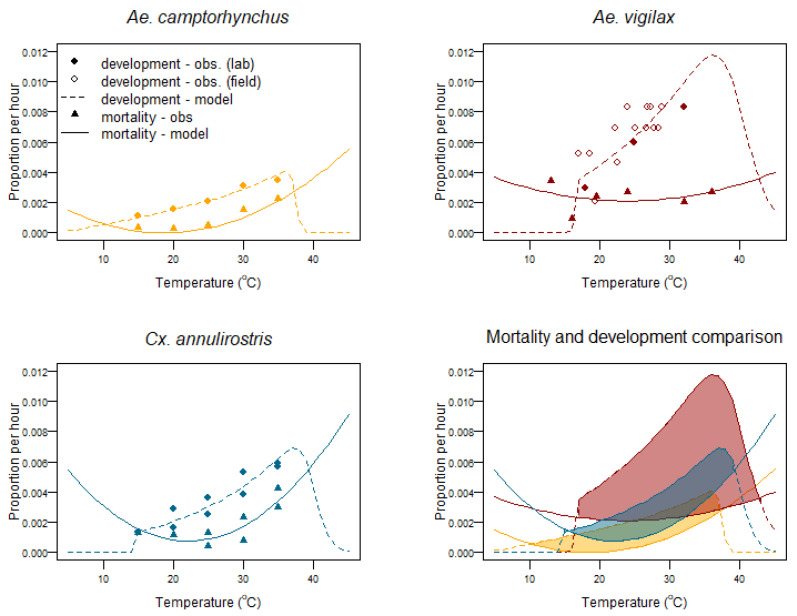
Proportion of mortality and development per hour for *Aedes camptorhynchus*, *Aedes vigilax*, and *Culex annulirostris* larval and pupal stages vs. water temperature—observed values vs. models.

The larval/pupal population is modelled by using the existing population, plus the number of newly hatched eggs, minus deaths due to thermal mortality. Larval/pupal development for all species is modelled using Equation (6), as shown in Figure 6. 

Development and mortality for *Cx. annulirostris* is based on the laboratory work of [58]. For *Ae. vigilax* field-based values are determined from a previous study in which only the air temperature is reported [57]. To estimate water temperature, historical meteorological records for Deception Bay [76] were obtained, and a mean value of 4 °C above the air temperature as the water temperature was used to estimate daily development proportions. Low and high temperature development limits were set at 16 °C and 45.5 °C, respectively. The low temperature threshold is set as larvae are not observed below this temperature [43]. The high temperature development threshold is set at 45.5 °C and is based on one-hour Thermal Death Points for larvae of *Cx. annulirostris* [80] and *Ae. aegypti* [79,83] as it is a species that also develops at high temperatures.

*Ae. camptorhynchus* development has not been studied at high or low temperature limits. A low temperature development threshold of 7.3 °C has been previously estimated [55], so this is used as the low temperature limit; the high temperature development limit is set at 40 °C, as its larval/pupal development peaks at a lower temperature than *Ae. aegypti* [84]. 

Mortality for all three species was modelled as a quadratic relationship fit to the observed data for *Ae. camptorhynchus* and *Cx. annulirostris*. This is likely to be an overestimation of high temperature survival, especially for *Cx. annulirostris*; however, the increased mortality at temperatures over 35 °C curtails successful development, see Figure 6. No studies have been conducted on *Ae. vigilax* larval mortality, so observations for *Ae. taenirohynchus* [52] were used, Figure 6.

#### 2.2.5. Adult Development and Mortality

The male to female emergence rate is 1:1 for all species. A 183/201 male to female emergence rate for *Cx. annulirostris* has been reported [53]; however, this is not a statistically significant difference (z-test, *p* = 0.359).

**Figure 7:**
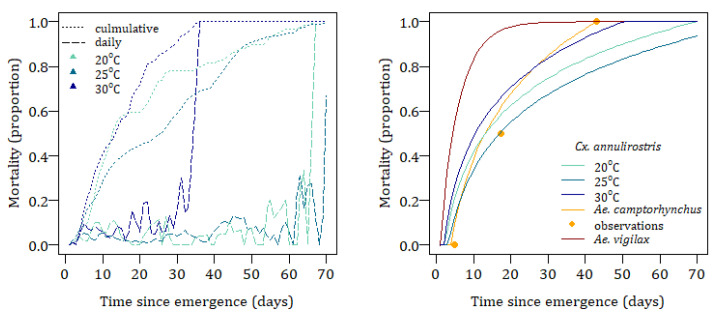
Observed adult daily and cumulative mortality for *Cx. annulirostris* (**left**). Modelled cumulative mortality for *Aedes camptorhynchus*, *Aedes vigilax*, and *Culex annulirostris* by time since emergence (**right**).

The longevity of *Cx. annulirostris* has been shown to vary both by the age of the mosquito [58], as shown in Figure 7, and air temperature and is modelled as the mean cumulative temperature, $T_{culm}$, since the time the adult emerged. The adult mortality probability, $M_{A}$, at time, t, in hours is given by Equation (14) where the functions $f\left(T_{culm}\right)$ and $g\left(T_{culm}\right)$ are determined by curve fitting.

(14) $$M_{A}=f\left(T_{culm}\right)+g\left(T_{culm}\right)\times ln\left(t/24\right)$$

To account for the ability of adult females to enter a state of quiescence, maximum life expectancy is set at 70 days when the mean weekly air temperature is below 17 °C. This temperature is chosen as it is generally the temperature at the study site between May and October each year, when the species is generally found to be present but inactive in similar climates in Australia [61,85].

The longevity of *Ae. camptorhynchus* is age dependent, Equation (15), and is estimated using the observations for this species’ survival in the laboratory at 20 °C [53], and upper thermal limits are set based on observations for *Ae. albopictus* of 50% and 90% mortality at 35 °C and 37.5 °C, respectively [86]. No estimates are available for *Ae. vigilax* lifespan so they are modelled on the daily survival estimate of 0.178 for *Ae. aegypti* [86]. Mortality curves for all three species are shown in Figure 7.

(15) $$M_{A}=0.421\ln\left(t\right)-0.584$$

##### Gonadotrophic Time 

Adults are assumed to have unlimited access to blood-meal hosts, so blood feeding and gonadotrophic development are modelled as a single stage, called gonadotrophic development, $D_{G}$.

Gonadotrophic development is temperature dependent. Development times for each species are given in Table 2. There is limited information about the temperature relationship with gonadotrophic development, so a linear model was fitted such that gonadotrophic development per hour, $D_{G}$, is a function of mean air temperature, $T_{airmean}$, fitted to species observations, as in Equation (16).

(16) $$D_{G}=AeT_{airmean}+B$$

##### Egg Laying and Egg Batch Size

*Ae. camptorhynchus* lay eggs at the edge or on the water surface, which can then float to the edge of the pool and lodge in the mud, or sink to the water bottom [53] within the preferred vegetation complex [41]. *Ae. vigilax* have been observed to prefer damp areas in the same habitat. In the model, eggs are laid at the current water height, $W_{H}\left(i\right)$, plus a random variable with SD 50 mm above the water level, Equation (17).

(17) $$H_{L}(i)=W_{H}\left(i\right)+|N\sim \left(0,50\right)|$$

If one pond is dry when a female is ready for oviposition, all eggs are laid around the pond containing water. If both ponds are dry the eggs are considered to be either not laid, or to not have sufficient moisture to be viable and so are removed from the model.

*Cx. annulirostris* females lay egg rafts on the water surface, if the water level is zero, they do not survive. The egg batch size for *Cx. annulirostris* is modelled as a quadratic equation of mean water temperature fitted to observed values which have a peak of around 260 eggs at 27.5 °C and decrease at lower and higher temperatures [59]. Egg batch sizes for the two *Aedes* species are drawn from a normal distribution with no temperature dependence.

## 3. Results

**Figure 8:**
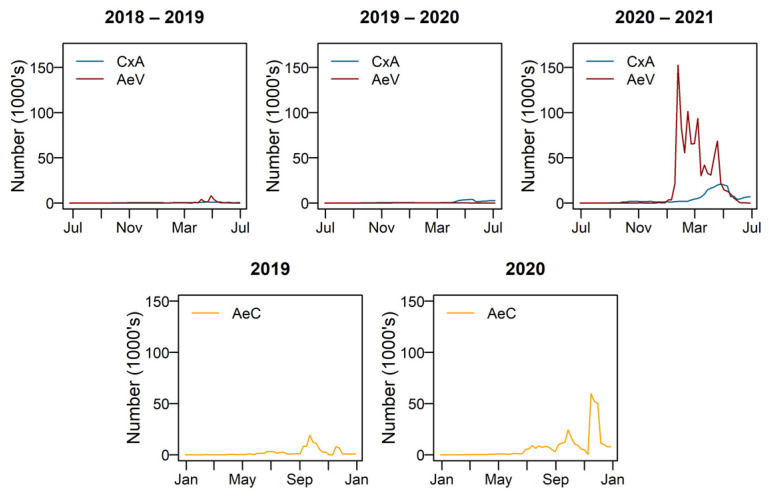
Modelled number of adult females over time. CxA = *Culex annulirostris*, AeV = *Aedes vigilax*, AeC = *Aedes camptorhynchus*.

The model was run for 2018–2019, 2019–2020 and 2020–2021 for *Culex annulirostris* and *Aedes vigilax,* which are most active during summer, and 2019 and 2020 for *Aedes camptorhynchus* which remains active in winter and early spring. Figure 8 shows the modelled number of adult females emerging for each species for each of the simulated years. 

### 3.1. Culex annulirostris

**Figure 9:**
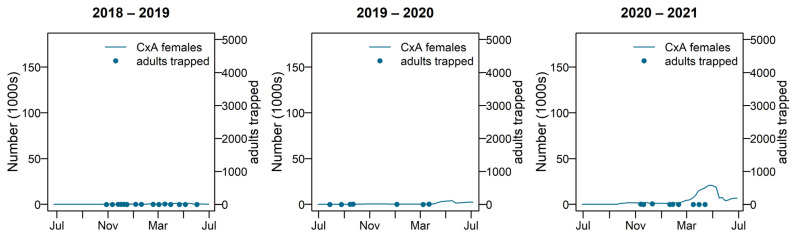
Adult female *Culex annulirostris* numbers for 2018–2019, 2019–2020, and 2020–2021; shown with adult trapping results plotted on the left-hand side y-axis. Cx A = *Culex annulirostris*.

The predicted number of adult female *Cx. annulirostris* remained low during 2018–2019 and 2019–2020. Overall, 2020–2021 is a peak year for species numbers, and 2018–2019 and 2019–2020 were less productive. Figure 9 compares the modelled number of adult females with the number caught during adult trapping. 

### 3.2. Aedes camptorhynchus

**Figure 10:**
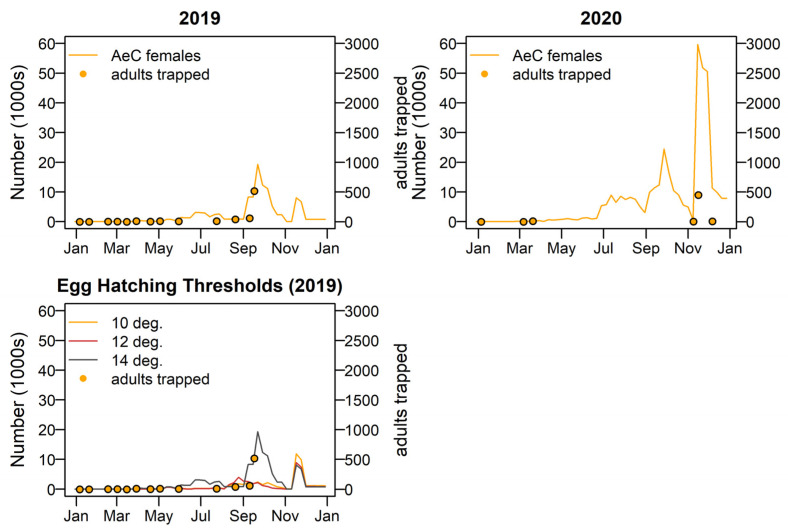
Adult female *Aedes camptorhynchus* numbers for 2019 and 2020; shown with adult trapping results plotted on the left-hand side y-axis. Three different egg hatching threshold temperatures were tested, bottom left.

*Aedes camptorhynchus* show a pattern of large peaks in spring and a smaller peak in late autumn each year, with 2020–2021 being the most productive. Adult trapping numbers agree well with modelled numbers of *Ae. camptorhynchus*. The best model fit was when a hatching threshold temperature of 15 °C was applied. The year 2019 showed relatively low levels of activity and 2020 relatively higher activity. Peak adult populations occur in early and late spring in both years, as shown in Figure 10.

### 3.3. Aedes vigilax

**Figure 11:**
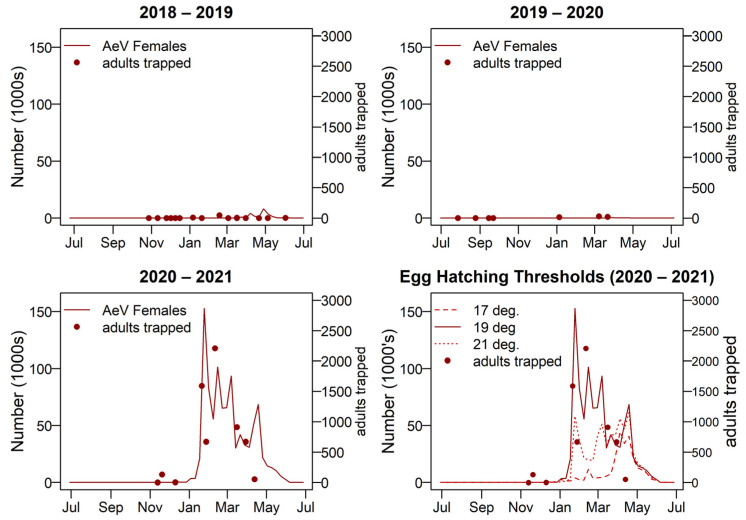
Adult female *Aedes vigilax* numbers for 2018–2019, 2019–2020, and 2020–2021 seasons; shown with adult trapping results plotted on the left-hand side y-axis. A comparison of adult female numbers with three egg hatching threshold temperatures is also shown, bottom right. AeV = *Ae. vigilax*.

*Aedes vigilax* show a large summer peak in 2020–2021 and not much activity in 2018–2019 or 2019–2020. When using the linear model of egg mortality *Ae. vigilax* became extinct within the first generation in 2018–2019 and 2020–2021. In 2019–2020 it ran for four generations and the egg bank remained relatively stable, starting with 24,000 eggs and ending with approximately 20,000. Using the proportional model of egg mortality no extinction occurred, and the resulting adult numbers are shown in Figure 11. Overall, using the hatching threshold temperature of 19 °C gave the most alignment with adult trapping records so was used for the model outputs shown. 

#### Egg Bank 

**Figure 12:**
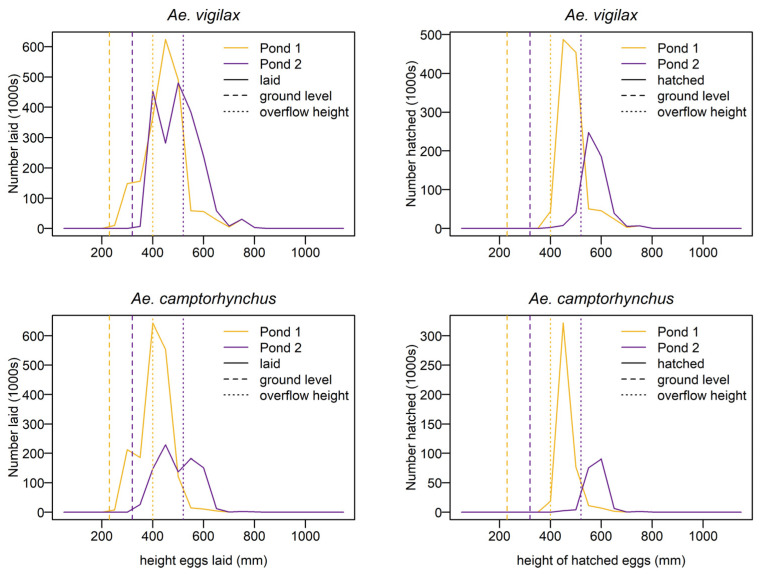
Distribution of the heights at which eggs were laid (**left**) and hatched (**right**) for *Ae. camptorhynchus* (**bottom**) and *Ae. vigilax* (**top**) eggs.

The mean height of eggs laid is well above the base level of the waterbody and close to the overflow riverbank level for both mosquito species. The mean height of eggs that eventually hatch is higher than the mean height of eggs laid for both species, indicating that eggs laid higher in the landscape but within the reach of tidal inundation have a greater survival probability. The distribution of eggs laid and hatched by height is given in Figure 12. This shows the waterbody with the lower overflow threshold produces the highest number of hatched eggs for both species. A summary of the distribution of the eggs laid and hatched by height for each waterbody is shown in Table 3.

## 4. Discussion

The model responds well to differing environmental parameters. All mosquito species showed relatively higher populations in the final year modelled but with intra-species differences. This shows the model is sensitive to species-specific parameters. Overall, the season of 2020–2021 supported higher levels of female emergence. This is reflected in the number of field sampled adults. That year was characterized by significantly more frequent tidal inundation, especially in summer, and higher spring rainfall, more than twice as much as the previous two spring seasons. Maximum and minimum air temperatures were similar across all three years. Both waterbodies were dry for more than 12 h on 8 days in 2020–2021, as compared to 33 and 54 days in 2019–2020 and 2018–2019, respectively.

### 4.1. Culex annulirostris

The output shows *Cx. annulirostris* do not breed prolifically in this environment during the peak summer period despite being considered a summer breeding species. In the Autumn of 2020–2021 their numbers accumulated to a certain extent. This agrees with the previous observations by [57], and Cooling, 1923b in [32] that *Cx. annulirostris* displace *Ae. vigilax* towards the end of the season. A physical explanation for this lies in the high mortality proportion at temperatures above 33 °C than for *Aedes vigilax*, see Figure 6. The high daily temperature fluctuation of an exposed shallow water environment regularly places them outside of their higher temperature development limit. Their larval mortality at higher temperatures would suggest that they would survive better in a deeper water environment which does not have such large daily temperature fluctuations. This is supported by the observations of [88] who showed that when given a choice of three water depths *Cx. annulirostris* lay egg rafts preferentially in deeper water, with over two thirds of rafts being laid in water 100 mm deep. *Culex* in the field have also been shown to prefer deeper water relative to *Aedes* species [25,89]. The modelled increase in *Culex* adult numbers was not reflected in the adults trapped at the end of the 2020/2021 season. This could be due to the effects of the long-term s-methoprene application that season, which had been in place since September 2021. It is also likely that temperatures from April onward are cooling and adults during this period become less active, possibly entering quiescence or diapause, as they head into their overwintering period. CO_2_ light trap counts can be negatively affected by local environmental conditions such as excessive wind, rain, or temperatures below their lower activity threshold. CO_2_ traps attract females looking for a blood meal and catch only a portion of those nearby. A previous study found only 13–16% of approaching females were captured by the trap [90]. Thus, this trap bias could explain the reduction in the observed adult females in the surrounding area which would be expected to be higher. 

Overall *Cx. annulirostris* population numbers are limited at this site, but the site conditions will change as climate change increases sea levels and temperatures [88], particularly if the site becomes more frequently inundated and capable of supporting floating aquatic macrophytes which can be an ovipositional preference for this species [89]. If water depth increases under these scenarios, *Cx annulirostris* is likely to become a more dominant vector species in this region.

### 4.2. Aedes camptorhynchus

It has been theorised that a cool, wet spring with occasional very high tides is more likely to produce large numbers of *Ae. camptorhynchus* [18]; however, the results of this study were that *Aedes camptorhynchus* had a relatively larger population in the spring of 2020 than in 2019. Overall, 2020 had more frequent and higher tidal inundations, so this does not support this view, although longer-term analysis would be required to be definitive. It was also proposed that *Ae. camptorhynchus* larval populations are driven significantly by rainfall [18], although this was subsequently disproved for southern Western Australia by [90]. The output of this model also shows that rainfall is not a significant driver of larval population in the study area. This highlights the importance of applying models to local conditions as small changes in soil type, rainfall, or elevation can result in large differences in larval populations. 

There is a lot that is yet unknown about this species including its development, survival, hatching thresholds at lower temperatures, upper thermal mortality limit, if there is a variation in egg batch size with temperature, how the adult lifespan may vary with temperature, and whether installment hatching may vary with the environmental. For example, eggs of *Ae. albopictus* laid under shorter photoperiods may hatch relatively later in the following season [91], and *Ae. albopictus* may undergo diapause in the adult stage and the egg stage in temperate environments [84]. Overall, the model is very useful for predicting peaks in *Ae. camptorhynchus* abundance at the field site, and the relative magnitude of those peaks across years. 

### 4.3. Aedes vigilax

The model for *Ae. vigilax* showed strong agreement with the adult trapping record overall, with limited breeding in 2018/2019 and 2019/2020, and a large population peak in 2020/2021. Being able to populate in high numbers in high summer temperatures and in a shallow water environment show *Ae. vigilax* has an ability to exploit an ecological niche in which other mosquito species struggle to survive. This species is very sensitive to the lower egg hatching threshold. Determining what egg conditioning is required for hatching is very important, and a few degrees can change the pattern of emergence for the entire year. When the hatching threshold was set at 17 °C the number of females in early summer was low. When the threshold was 21 °C the number of females in early summer was too low and in late summer was too high. It was estimated that egg conditioning of a mean weekly air temperature of approximately 19 °C may be what occurs in this species, but this needs to be confirmed with further research.

Contrary to the idea that *Ae. vigilax* are more likely to occur in large numbers during periods of high temperature and low tides [13], for 2020–2021 Figure 2 shows that the frequency and heights of the tides in summer were greater than the previous 2 years and these proved conducive to large populations of *Ae. vigilax*. This species certainly requires the high temperatures of summer to thrive in large numbers, but frequent inundation also results in large populations. However, if inundation increased to the point of becoming permanent, it is possible fish and other predators of this species may become established and reduce the overall numbers of *Ae. vigilax* emerging from the site.

Further research into egg longevity at different temperatures is required for this species, as this can have a marked impact on survival at specific sites. Under the linear model, this species became locally extinct in the first half of 2018/2019. Extending the lifespan slightly allowed the species to survive. It is possible that local extinction occurs, and repopulation is from nearby areas with more favorable conditions; however, it would seem likely that nearby surrounding areas experience similar conditions as they are flooded by the same water source with a very similar frequency.

#### Egg Bank Height

Under the conditions of the model, both *Aedes* species lay eggs at around the waterbody overflow height, as shown in Table 3. This agrees with previous research finding higher numbers of *Ae. vigilax* eggshells at the edges of ponds and depressions and relatively fewer at the bottom of ponds [81,92]. It also supports the finding that *Ae. camptorhynchus* distribution within saltmarsh environments was more related to vegetation than elevation [41]. This is consistent with the modelled egg distribution as samphire vegetation is found on the edges of pools and surrounds, rather than the bottoms. The mean hatching height for both species was higher and more dispersed, as indicated by the larger standard deviation, indicating that higher egg laying height increases the probability of successful hatching, with the higher elevation limit being the frequency of high tides being less than the lifespan of the egg.

## 5. Conclusions

The model will be useful for examining the effect of different seasonal patterns and other possible impacts on the abundance of these species but is limited by the relatively small number of studies on the physiology of these species. Other major gaps in knowledge are the egg hatching cues such as the minimum temperature thresholds, egg development rate, gonadotrophic time and adult survival at different temperatures for the *Aedes* species, and the larval development and mortality rates at the limits of their temperature range, particularly the high-end limit as that will become more relevant as the climate changes.

After further testing and validation of this model across a range of sites, it could be used to provide insight into different treatment regimens, predicting the impact of treatment of different periods or over different areas. It could also be coupled with a spatial heterogeneous adult dispersal model, to further explore disease transmission dynamics, or be coupled with remote water-height sensing in regional areas to assist in mosquito control programs in locations with many waterbodies that require monitoring but where there is limited resourcing to do so. This model can also be used to investigate the changes to mosquito species diversity and abundance at a local scale under different climate change scenarios.

## Figures and Tables

**Table 1:** Egg stage parameters.

Attribute	Species	Value	Reference
Egg hatch mortality	*Ae. camptorhynchus*	2%	[42]
	*Ae. vigilax*	17%	[40]
Egg maximum lifespan	*Ae. camptorhynchus*	15 months	[42]
	*Ae. vigilax*	116 days at 65% RH 98 days at 17% RH	[43]
Egg density/m^2^ (initial)	*Ae. camptorhynchus*	0.24 SD 0.05	[81]
Embryonic development time	*Ae. vigilax*	48–54 h	[49]
	*Cx. annulirostris*	1.25–5 days at 35 °C to 15 °C	[58]
Instalment hatching rate	*Ae. camptorhynchus*	43%	[53]
	*Ae. vigilax*	from 0% at 8 °C to 98% at 11.5 °C	[43]

**Table 2:** Adult mosquito parameters.

Attribute	Species	Value	Reference
Gonadotrophic time	*Ae. vigilax*	72–96 h	[43,50]
	*Ae. camptorhynchus*	5–21 days	[54]
	*Cx. annulirostris*	4–9 days	[43]
Daily mortality	*Ae. vigilax*	0.178 (*Ae. aegypti)*	[87]
	*Ae. camptorhynchus*	17.4 days at 20 °C, 5–43 days	[54]
	*Cx. annulirostris*	age & temperature dependent	[43,60]
Egg batch size	*Ae. vigilax*	N~(69.3, 19.8)	[88]
	*Ae. camptorhynchus*	N~(64, 18)	[54,55]
	*Cx. annulirostris*	100–260	[58]

**Table 3:** Egg bank height characteristics for *Ae. vigilax* and *Ae. camptorhynchus*.

	Base Level (mm)		Overflow Height (mm)		$\overset{-}{x}$ Height Laid (mm)		$\overset{-}{x}$ Height Hatched (mm)	
Mosquito Species	Pond 1	Pond 2	Pond 1	Pond 2	Pond 1	Pond 2	Pond 1	Pond 2
*Ae. camptorhynchus*	230	320	400	520	497 SD 77	506 SD 73	464 SD 40	578 SD 39
*Ae. vigilax*	230	320	400	520	449 SD 84	501 SD 81	486 SD 54	573 SD 49

## Data Availability

Publicly available datasets were analyzed in this study. Tide height data was obtained from the Department of Transport and are available from https://www.transport.wa.gov.au/imarine/download-tide-wave-data.asp (accessed on 19 November 2021). River height data was obtained from the Department of Transport and are available from https://www.transport.wa.gov.au/imarine/download-tide-wave-data.asp (accessed on 19 November 2021). Restrictions apply to the availability of weather data, which was obtained from the Bureau of Meteorology and are available from http://www.bom.gov.au/climate/data-services/data-requests.shtml (accessed on 21 November 2021) with the permission of the Bureau of Meteorology. Restrictions apply to the availability of mosquito monitoring data, which were obtained from the Town of Bassendean https://www.bassendean.wa.gov.au/your-town/work-with-us/contact-us.aspx (accessed on 21 March 2017). R code is available from the corresponding author upon request. All remaining relevant data are within the paper.
